# The Effect of Acute Caffeine Ingestion on Cognitive Dual Task Performance during Assessment of Static and Dynamic Balance in Older Adults

**DOI:** 10.3390/nu12123653

**Published:** 2020-11-27

**Authors:** Jason Tallis, Chelsea Bradford, Michael J. Duncan, Sheila Leddington-Wright, Matthew F. Higgins, Matthew Hill

**Affiliations:** Centre for Applied Biological and Exercise Sciences, Alison Gingell Building, Coventry University, Priory Street, Coventry CV15FB, UK; walshc9@uni.coventry.ac.uk (C.B.); aa8396@coventry.ac.uk (M.J.D.); ssx398@coventry.ac.uk (S.L.-W.); matt_higgins@hotmail.co.uk (M.F.H.); ab2225@coventry.ac.uk (M.H.)

**Keywords:** caffeine, balance, older adults, dual-task processing

## Abstract

The present work aimed to evaluate the effect of 3 mg·kg^−1^ caffeine consumption on the standing and dynamic balance performance of older adults and sought to establish if caffeine ingestion can modulate the influence of a cognitive dual task on balance performance. Twelve apparently healthy participants (8 females) aged >65 years (72 ± 3.7 years) completed the study. Bipedal postural sway, four square step test, timed up and go, Y-balance (anterior reach only) and force-time characteristics of sit-to-stand performance were used to assess standing and dynamic balance. Attention and working memory were assessed using a serial 3s and 7s subtraction task during seated rest and completion of the bipedal standing assessment and Y-balance test. This battery of assessments was completed on two separate occasions, once following the consumption of a non-ergogenic placebo and again following the consumption of 3 mg·kg^−1^ caffeine. The administration of treatments was randomised, counterbalanced and double-blind. Caffeine reduced performance in the bipedal standing balance assessments, evidenced by an increase in COP_ML_, COP_Path_, COP_Velocity_. Performance during the dynamic balance tests was unaffected, other than rate of force development during the sit-to-stand, which was improved following caffeine ingestion. The introduction of a cognitive dual task had either limited effects, or improved facets of bipedal standing balance, whilst performance during the dynamic balance task was significantly reduced. In both balance assessments, there was evidence for a reduction in the performance of the cognitive task when both the balance and cognitive tests were performed simultaneously, with this effect not modulated by caffeine consumption. These findings refute the idea that caffeine ingestion may have positive effects on balance performance. However, despite a caffeine-induced reduction in bipedal standing balance, it is unlikely that caffeine ingestion would exacerbate fall risk given the limited effects in the dynamic balance tests. Future work should establish if these effects are generalisable to older frail participants and if caffeine can modulate the detrimental effects of an acute exercise bout on balance performance.

## 1. Introduction

Caffeine is one of the most commonly consumed drugs in the world and has been studied extensively for its performance-enhancing effects [1,2,3,4,5,6]. There is a growing body of evidence indicating that acute caffeine ingestion may be effective for inducing improvements in endurance [1,7], muscular strength [5,6], high intensity exercise performance [8] and cognitive function [9]. For the most part, previous scientific evidence examining the effects of acute caffeine consumption focus on athletic performance in young adults. However, if such responses are apparent across the age span, acute caffeine ingestion could have profound benefits for the physical performance of older adults. One particular area of focus for older adults is balance, an important risk factor associated with falling [10]. Falls are a major public health concern, particularly in those aged over 65 years [11], with evidence indicating that falls represent the leading cause of accidental death in older adults [12]. Given the potential for caffeine to evoke improvements in both the physical components and cognitive process needed for effective balance, caffeine consumption may be an effective nutritional strategy to improve the balance performance of older adults.

Lower limb muscular strength has been demonstrated to be an important physical characteristic for effective balance [13,14] and the age-related reduction in strength is associated with increased fall risk [15,16]. There is now a strong body of evidence demonstrating that acute caffeine ingestion can elicit increased muscular strength [5,17,18], and this has been demonstrated to occur more specifically in older adults [19,20]. However, this finding is not consistent throughout the literature [21,22]. Consequently, improvements in muscular strength evoked by an acute dose of caffeine may transfer to improving the balance and physical function of older adults.

To date, research examining the effect of caffeine ingestion on balance is limited, specifically focused on young adult populations, with little commonality in findings between published works. Previous evidence indicates that acute caffeine ingestion may improve [23], decrease [24] or have little effect [25,26] on the standing balance performance of young adults. Such ambiguity may be in part related to variation in the administered caffeine dose, ranging from 160 to ~400 mg. Only Kara et al. (2018) provided a caffeine dose relative to body mass (6 mg·kg^−1^), and this was the only study to see beneficial effects. Furthermore, methodological inconsistences and variations in the balance tasks and outcome measures utilised could also explain the inconsistency in findings. Enriquez, Sklaar, Viirre and Chase [25] assessed balance using a bipedal standing task, reporting centre of pressure (COP) path length and permutations in both medial-lateral and anterior-posterior planes, whilst Liguori and Robinson [26] used a similar assessment but only derived balance performance from the COP displacement in the anterior-posterior plane. Kara, Patlar, Stoffregen and Erkmen [23] is the only study to assess the effect of caffeine using an assessment of dynamic balance, and unlike the previous studies, it reports overall stability index, although the specifics of how this is calculated were not reported. This limited evidence and the discrepancy in findings indicate a need for further and more comprehensive assessment of caffeine effects on balance performance.

To date, only two studies have assessed the effects of acute caffeine ingestion on the balance performance of older adults, and such work is limited to assessments of bipedal standing balance. Swift and Tiplady [27] demonstrated that COP amplitude in the anterior-posterior direction was significantly increased in six male and female participants aged 65–75 years, following the ingestion of 200 mg of caffeine, although such effects were only seen 3 h post ingestion. Similarly, more recent work by Norager, et al. [28], using a population of 30 males and females aged greater than 70 years, indicated that 6 mg·kg^−1^ caffeine resulted in greater velocity moment (mean area covered by the movement of the centre of pressure during each second of the test) in both eyes open and eyes closed conditions. Despite this evidence providing an important initial insight into the effects of caffeine on balance performance in older adults, this work is focused only on selected measures of bipedal standing balance. One important measure with respect to standing balance performance in older adults is medial-lateral sway, given its closer association with fall risk [12]. The effect of acute caffeine ingestion in older adults on medial-lateral sway during bipedal standing balance is yet to be determined. Furthermore, associations between standing and dynamic balance outcomes among older adults are generally weak [29], implying that the neuromuscular mechanisms governing static and dynamic/functional balance tasks differ. The effect of caffeine ingestion on the dynamic balance performance of older adults is yet to be examined. This is particularly important given that falls generally occur during locomotor tasks rather than during quiet standing [30]. Given that dynamic balance assessments involve elevated physical demands (i.e., greater muscular strength requirement) beyond those of quiet standing balance [31], there is a reasonable theoretical basis that caffeine may differentially effect static and dynamic balance components.

In order to more closely simulate the challenges to postural control that occur during daily living, the present study sought to further examine if acute caffeine ingestion can modulate the effects of a cognitive dual task on balance performance. Previous work has indicated that the completion of a secondary task can impair [32] or improve postural control but at the expense of the secondary task [33]. Furthermore, cognitive dual-tasking may become increasingly impaired with age [34,35], limiting mobility and increasing the risk of falls [36]. The beneficial effect of caffeine on aspects of cognitive function has been well established [9] and thus may harness the potential to modulate the influence of a cognitive dual task of postural control. This is yet to be explored in an older adult population.

As a result of the gaps in the knowledge base, the present work aimed to evaluate the effect of 3 mg·kg^−1^ caffeine consumption on the standing and dynamic balance performance of older adults and sought to establish if caffeine ingestion can modulate the influence of a cognitive dual task on balance performance. It was hypothesised that caffeine would increase postural sway during quiet standing but performance would be improved in dynamic balance tasks. Such changes in balance performance would not come at the expense of performance in cognitive tasks.

## 2. Method

### 2.1. Participants

Following ethics approval (P70909) and written informed consent, 12 apparently healthy male and female participants (8 females) aged >65 years (age: 72 ± 4 years; height: 164 ± 7 cm; body mass: 66.7 ± 14.0 kg) volunteered to participate in the study. Exclusion criteria included those suffering from a musculoskeletal injury that would prevent completion of the experimental procedures, inner ear disorders, cognitive impairment, those consuming psychoactive medication or suffering from un-medicated hypertension. Participants were habitual caffeine consumers (235 ± 162 mg per day), two of which were heavy caffeine consumers (i.e., >350 mg per day), as determined by a caffeine consumption questionnaire [37]. Participants also reported high self-perceived balance confidence (95.7 ± 9.5%) as measured by the Activities-Specific Balance Confidence Scale (ABCS) [38].

### 2.2. Experimental Procedures

Participants attended the human performance laboratory at the host institute on three occasions, and on each visit, they completed a departmental health screen questionnaire to determine suitability to complete the planned assessments. The intention of the first visit was to familiarise participants with the experimental procedures. Initially, height and body mass were measured via a stadiometer (SECA Instruments Ltd., Hamburg, Germany) and electronic weighing scales (SECA Instruments Ltd., Hamburg, Germany), respectively. Each individual then completed a caffeine consumption questionnaire [37], Brunel Mood Scale (BRUMS) [39] and the ABCS [38] to determine typical caffeine consumption habits, mood and perceived balance ability, respectively.

Following a period of 5 min of seated rest and remaining in a seated position, participants completed two serial subtraction tasks in which participants were asked to count backwards in 7s (serial 7s) and then again in 3s (serial 3s) for a period of 30 s. Participants then completed the Eriksen Flanker task. Bipedal standing balance was assessed in the manner outlined below and then repeated whilst completing both serial subtraction tasks. Following a short rest, participants were asked to complete the lower quartile Y-balance test (YBT-LQ), and then again during the completion of the serial subtraction tasks. Sit-to-stand, timed up and go (TUG) and four square step test (FSST) were then completed in the manner described below. The Eriksen Flanker Test, ABCS and BRUMS were reassessed upon completion of the balance tasks.

Participants then returned a minimum a minimum of 72 h following the familiarisation to complete two experimental trials. Prior to each of these visits, participants were advised to abstain from intense physical activity and caffeine consumption for 48 h and 12 h, respectively. The period of caffeine abstinence is unstandardised in the literature and is typically between 48 and 12 h prior to completion of the experimental trials [40,41,42]. A 12 h period of caffeine abstinence, equivalent to the evening prior to completing the exercise, was chosen, in line with previous work [40,43]. The period was deemed appropriate to optimise compliance and mitigate potential negative effects of caffeine withdrawal [44]. Participants attended the laboratory forty-five minutes prior to completion of the experimental tasks and consumed a capsule containing either caffeine (3 mg·kg^−1^ body mass) or an equivalent dosage of maltodextrin as a non-ergogenic placebo, with 4 mL/kg^−1^ body mass of water. Moreover, 3 mg·kg^−1^ body mass (~200 mg) was chosen as it was largely considered the lowest dose required to elicit and improvement in physical performance [45]. Furthermore, this dose more closely represents the typical caffeine consumption habits of older adults [46] and represents a quantity which would be easier to achieve from commercially available products without specifically seeking anhydrous caffeine. Treatments were administered in a double blind, randomised and counterbalanced fashion. Participants completed the serial 3s, serial 7s and Erikson Flanker test in a seated position followed by the ABCS and BRUMS both immediately pre-ingestion, 45 min post-ingestion and again following completion of the balance assessments. Assessments of standing and dynamic balance occurred in the manner previously described.

Experimental trials were separated by a minimum of 72 h. All testing took place between 10:00 and 14:00, with each condition taking place at the same time of day to control for effects of circadian variation on the performance measures [47]. Specific nutritional advice was not provided; however, participants were instructed to consume the same diet on the day of each trial.

### 2.3. Balance Assessments

#### 2.3.1. Standing Balance

To examine postural sway during upright bipedal stance, each participant stood barefoot on a single tri-axial force plate (AMTI, AccuGait, Watertown, MA, USA) sampling at a frequency of 100 Hz and completed the following four conditions: eyes open (EO), eyes closed (EC), eyes open whilst standing on compliant foam (EOF), eyes closed whilst standing on compliant foam (ECF). These combinations of sensory modulation (i.e., foam surface, eyes closed) have been shown to increase the level of difficulty of standing balance tasks, as deduced by varying degrees of postural sway and muscle activity [48]. Participants completed one practise and three recorded trials per condition. Trials lasted 30 s and were separated by at least 30 s of rest. Participants were instructed to step onto the force plate with feet shoulder width apart in an erect standing position. Participants were instructed to stand as still as possible for 30 s, with sway data recorded for the final 25 s of each trial. Participants were only instructed that the trial had ended following completion of data collection. During the EO condition, participants were asked to gaze at a circle, 10 cm in diameter, placed 1.8 m away at eye level. For each trial, the following parameters were measured: centre of pressure (COP) path length (cm) (COP_Path_), 95% elliptical area (cm^2^) (COP_Ellipse_), mean COP velocity (cm·s^−1^) (COP_Velocity_), maximal amplitude of the mediolateral (ML) (COP_ML_) and anteroposterior (AP) (COP_AP_) of the COP displacement (cm), which served as indicative measures of postural sway [49]. The average from the three parameters from each trial was used in subsequent analysis. The validity and reliability of these parameters have previously been established for this sampling duration [50].

#### 2.3.2. Y-Balance

The lower quartile Y-balance test (YBT-LQ) was completed in accordance with previous research [51], but for the anterior reach distance only. Our preliminary work indicated that a number of the participants that completed the current study were unable to safely complete posterior reach directions. The anterior reach direction was used to mimic dynamic balance used in stepping. Participants completed three trials standing on the dominant limb followed by three trials standing on the non-dominant limb. The trial that elicited the greatest reach distance was used for analysis.

#### 2.3.3. Sit-To-Stand

The sit-to-stand (STS) test is a well-used assessment tool for measuring functional mobility, leg muscle strength and speed of force development [52], which are key factors of functional movement in healthy ageing [53]. Sitting in a normal chair, participants were asked to stand as quickly as possible onto two uniaxial force platforms (Pasport, PASCO, US) sampling at 1000 Hz and maintain a stable standing position for 10 s. Participants performed three repetitions of the STS and the trial that yielded the greatest vertical force was used in the analysis. Peak vertical force (FBW), rate of force development (N/S) and time to stabilisation were determined. Rate of force development was calculated as (Peak vertical force (N)/(Time of peak force (s)—Time of initial movement (s)). Time to stabilisation was calculated as the time from initial movement until the first time point in which the vertical force trace returned within 2% of body weight for a period of at least 100 ms.

#### 2.3.4. Timed Up-And-Go

The TUG test is a simple yet commonly used test for evaluating lower limb muscle function, mobility and fall risk [54]. Participants completed an 8-foot TUG by moving from a seated position to standing, walking 8 feet, turning and returning to a seated position as quickly as possible. Time to complete the task was recorded using a video camera (Nikon, Coolpix B500) and analysed using Quintic Biomechanics version 26.0 (Quintic Consultancy Ltd., Birmingham, UK). Participants completed the task three times, with each attempt separated by 30 s of rest. Participants were instructed to complete the course as fast as possible and the fastest time was recorded and used in later analysis.

#### 2.3.5. Four Square Step Test (FSST)

The FSST was completed in accordance with published protocols [55]. Two runs of electrical tape 180 cm in length were placed on the floor, crossing at their mid-point at an angle of 90 degrees. The squares were numbered from 1 to 4, and starting in square 1, participants were asked to rotate clockwise around the quadrant, moving into each numbered squared with both feet. This was then reversed and participants moved around the quadrant counter-clockwise. The following instructions were given to the participant: “Try to complete the sequence as fast and as safely as possible without touching the tape. Both feet must make contact with the floor in each square”. Participants were allowed one practice and two timed trials, with performance time measured using a stop watch. If the tape was touched, the participant lost balance or failed to place both feet in the square, then the trial was repeated.

### 2.4. Cognitive Task

#### 2.4.1. Serial 3s and Serial 7s Task

Sequential subtracting tasks of serial 3s and serial 7s are commonly used tests of attention and working memory [56] and are widely used in dual-task paradigms [57,58]. Here, participants were asked to recite aloud serial subtractions of 7 or 3, starting from a random 3-digit number, for a period of 30 s. For the serial 7s and 3s task, a random start number was generated 591–597 and 291–297, respectively. Responses were recorded via a dictaphone and performance was marked for accuracy and speed.

Serial counting tasks were completed at rest both pre- and post-ingestion, during EO bipedal standing conditions and during the YBT-LQ. In each case, the assessment was standardised to last a fixed period of 30 s. Cognitive performance was reported as the number of responses per second, calculated as (number of responses)/30. Accuracy was determined via the calculation of a corrected response rate (CRR) using the following equation: response rate per second × percent correct.

#### 2.4.2. Eriksen Flanker Task

The Eriksen Flanker task [59] was used as a measure to examine the attentional control process [60]. The test requires a rapid response to a central target stimulus which is flanked simultaneously with two distractor stimuli. The test included exposure to both congruent trials, where the flankers were associated with the same response as the target stimuli (e.g., <<<<<), and incongruent trials, where the flankers were presented as a conflicting response (e.g., >><>>). Participants completed an online version of the test using a laptop computer (Sony Vaio, Sony Inc, Japan) via open source experimental software [61] and were instructed to respond according to the target, ignoring the flankers. Responses to stimuli were made using the keyboard. As per the method used in our previous work [40], participants were administered 100 trials, consisting of equiprobable congruency and directionality. Stimuli were 2.5 cm tall white arrows presented focally for 120 ms on a black background with a response window of 1000 ms and a variable inter-stimulus interval of 1100, 1300 or 1500 ms. Total task duration was approximately 3 min. Response speed and accuracy were then calculated for each of the congruent and incongruent trials.

### 2.5. Statistical Analysis

Normality and homogeneity of variance were tested using Shapiro–Wilk and Mauchly tests, respectively. COP_Ellipse_, COP_Path_, COP_AP,_ COP_ML_ and COP_Velocity_ variables determined from the standing and dynamic balance assessments, anterior reach distance determined from YBT-LQ, CRR during completion of the serial 3s and 7s task, mood and CRR and RT determined from the Flanker task were analysed using a series of treatment x trial repeated measure analyses of variance (ANOVA). Violations of sphericity were corrected using Greenhouse–Geisser where applicable. Where appropriate, Bonferroni-corrected pairwise comparisons were performed to identify differences between trials. On a small number of occasions, normality was violated; however, ANOVA was still considered a robust statistical method in such cases [62,63]. For ANOVA, partial eta squared (ηp^2^) was calculated as an estimate of effect size and should be interpreted as small (>0.01), medium (>0.06) and large (>0.14) [64]. Paired samples *t*-tests or Wilcoxon signed-rank tests were used to determine the effect of caffeine on the outcome variables determined from STS, FFS and TUG. For *t*-tests, Cohen’s d was calculated and corrected for bias using Hedge’s *g* [65]. For Wilcoxon signed-rank tests, effects size (*r*) was calculated as *Z*/√*n* [66]. Hedges *g* effect size should be interpreted as trivial (<0.2), small (<0.6), moderate (<1.2) or large (>1.2) [67], and for *r,* small (>0.1), medium (>0.3) and large (>0.5) [68]. Data are presented as mean ± S.E.M. Statistical analysis was performed using SPSS 26.0 (Chicago, IL, USA). Statistical significance was a priori set at an alpha level of *p* < 0.05. Graphical presentation was performed using GraphPad Prism (Version 8.3.1, San Diego, CA, USA).

## 3. Results

### 3.1. Standing Balance

**Figure 1:**
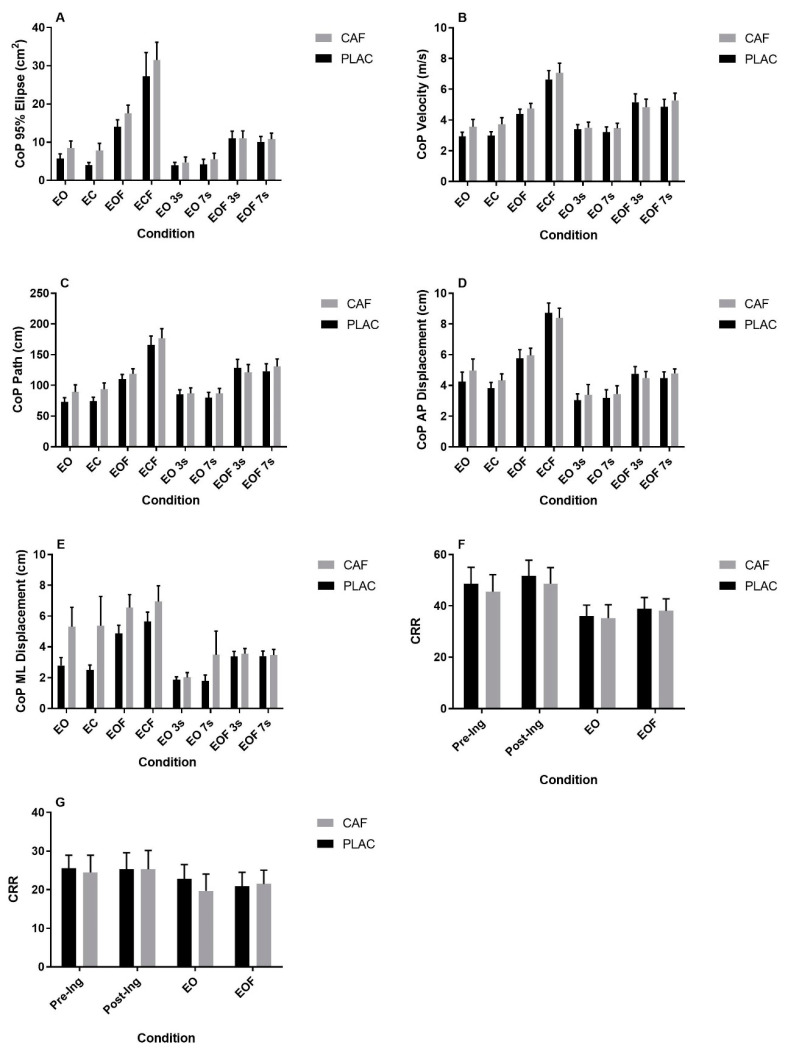
The effect of caffeine consumption on COP measures of postural sway (**A**–**E**) and serial 3s (**F**) and 7s (**G**) correct response rate (CRR) during completion of a 30 s static balance assessment. Data represented as mean ± S.E.M.; *n* = 12 in each case; COP = Centre of Pressure; EO = eyes open; EC = eyes closed; EOF = eyes open on foam; ECF = eyes closed on foam; EO 3s = eyes open completing serial 3s; EO 7s = eyes open completing serial 7s; EOF 3s = eyes open on foam completing serial 3s; EOF 7s = eyes open on foam completing serial 7s; CAF = Caffeine; PLAC = Placebo.

There were no significant treatment x trial interactions for any of the quiet standing COP measures (Figure 1, ANOVA *p* > 0.09; *ηp*^2^ < 0.12 in each case). Caffeine treatment had no effect on COP_Ellipse_, COP_AP_ (Figure 1A, C, ANOVA *p* > 0.152; *ηp*^2^ < 0.18 in both cases), but significantly increased COP_ML_, COP_Path_, COP_Velocity_ when compared to placebo (Figure 1B, C and E, ANOVA *p* < 0.05; *ηp*^2^ > 0.32 in each case). There was a significant effect of trial for all measures (Figure 1, ANOVA *p* < 0.001; *ηp*^2^ < 0.42 in all cases), with COP measures being significantly higher in the ECF trial in 28 of the 35 comparisons (Figure 1A–E, Bonferroni *p* < 0.05 in each case). Completion of the serial 3s and 7s task in the EO condition had no effect on COP measures compared to the EO only trial (Figure 1, Bonferroni *p* > 0.05 in each case). However, COP_Ellipse_ and COP_ML_ were significantly reduced in the serial 7s task when standing on foam compared to the EOF trial (Figure 1A,E, Bonferroni *p* < 0.05). There were no other significant differences (Figure 1A–E, Bonferroni *p* > 0.05 in each case).

CRR during the serial 3s task was not significantly affected by treatment (Figure 1F, ANOVA *p* = 0.340; *ηp*^2^ = 0.083), nor was there a treatment x time interaction (Figure 1F, ANOVA *p* = 0.881; *ηp*^2^ = 0.02). CRR was significantly affected by time (Figure 1F, ANOVA *p* < 0.001; *ηp*^2^ = 0.623). There was no difference in CRR pre-compared to post-ingestion (Figure 1F, Bonferroni *p* = 0.549); however, CRR was significantly lower during the completion of the balance tasks (Figure 1F, Bonferroni *p* < 0.031 in both cases). There was no difference in CRR between the EO and EOF conditions (Figure 1F, Bonferroni *p* = 0.803). CRR during completion of the serial 7s task was not significantly affected by treatment (Figure 1G, ANOVA *p* = 0.712; *ηp*^2^ = 0.013), time (Figure 1G, ANOVA *p* = 0.066; *ηp*^2^ = 0.210), nor was there a treatment x time interaction (Figure 1G, ANOVA *p* = 0.524; *ηp*^2^ = 0.065).

### 3.2. Dynamic Balance

**Figure 2:**
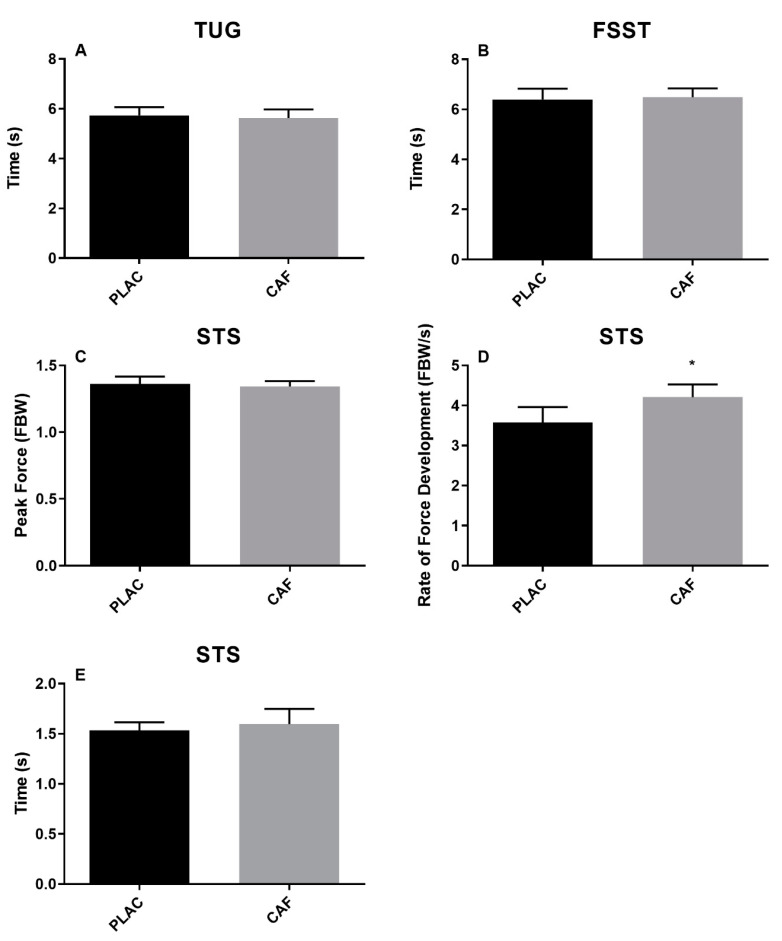
The effect of caffeine consumption on timed up-and-go (TUG) (**A**), four square step test (FSST) (**B**), and sit-to-stand (SST) performance (**C**–**E**). (Data represented as mean ± S.E.M.; *n* = 12 in each case; * indicates the difference between treatment at the alpha level *p* < 0.05; CAF = Caffeine; PLAC = Placebo).

TUG, STS peak force, STS time to stabilisation measured and time to complete FSST were not significantly affected by treatment (Figure 2, *p* > 0.38 in each case; *g* < 0.12; *r* < 0.25). However, STS RFD was significantly greater in the caffeine trial (Figure 2, *p* = 0.01; *g =* 0.44).

**Figure 3:**
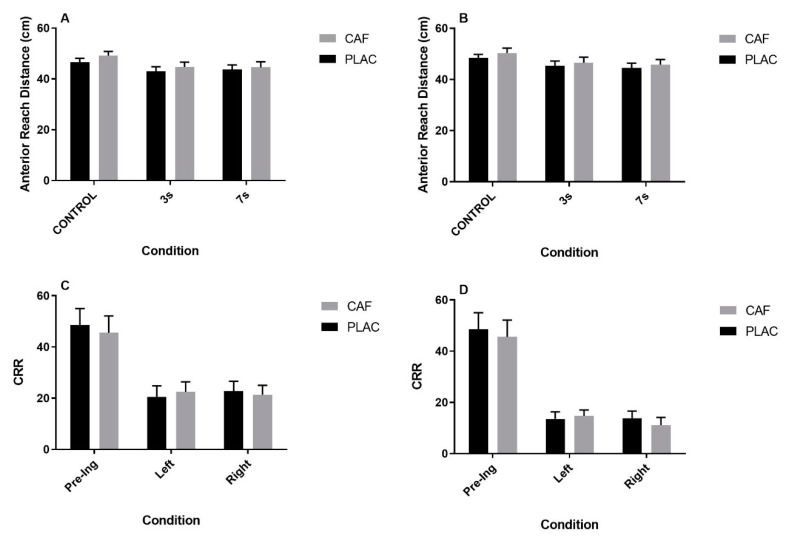
The effect of caffeine consumption on dominant (**A**) and non-dominant leg (**B**) anterior reach distance and serial 3s (**C**) and 7s (**D**) correct response rate (CRR) during the (lower quartile Y-balance) YBT-LQ test. Data represented as mean ± S.E.M.; *n* = 12 in each case; 3s = serial 3s; 7s = serial 7s; Pre-Ing = pre-ingestion; CAF = Caffeine; PLAC = Placebo.

Anterior reach distance for both the right and left legs during the YBT-LQ assessment was significantly affected by trial (Figure 3A,B, ANOVA *p* < 0.013; *ηp*^2^ > 0.416 in each case), although there was no main effect for treatment (Figure 3A,B, ANOVA *p* > 0.136; *ηp*^2^ < 0.191 in each case) nor a significant treatment x trial interaction (Figure 3A,B, ANOVA *p* > 0.228; *ηp*^2^ < 0.063 in each case). For both the dominant and non-dominant legs, anterior reach distance was significantly reduced during completion of the serial 3s task (Figure 3A,B, Bonferroni *p* < 0.016 in each case) compared to control. Anterior reach distance was also reduced during completion of the serial 7s task, but only for the non-dominant leg (Figure 3B, Bonferroni *p* = 0.014). There was no difference in the anterior reach distance achieved during the serial 3s trial compared to the serial 7s (Figure 3A,B, Bonferroni *p* > 0.926 in each case). Similarly, CRR during completion of the serial 3s and 7s task was significantly affected by trial (Figure 3C,D, ANOVA *p* < 0.009; *ηp*^2^ > 0.465 in each case), although there was no main effect for treatment (Figure 3C,D, ANOVA *p* > 0.389; *ηp*^2^ < 0.069 in each case) nor a significant treatment x trial interaction (Figure 3C,D, ANOVA *p* > 0.448; *ηp*^2^ < 0.071 in each case). For both the serial 3s and 7s, CRR during completion of both left and right YBT-LQ assessments was significantly lower compared to control (Figure 3C,D, Bonferroni *p* < 0.036 in each case). There was no significant difference in CRR between assessments performed on the left and right sides (Figure 3C,D, Bonferroni *p* > 0.85 in each case).

#### 3.2.1. BRUMS

There was no main effect for treatment or time or a treatment x time interaction for perceived anger, depression, fatigue, tension and vigour measured by the Brunel Mood State questionnaire (Table 1, ANOVA *p* > 0.11; *ηp^2^* < 0.212 in each case). Given that there was a treatment x time interaction for confidence (Table 1, ANOVA *p* = 0.049; *ηp^2^* = 0.240), a series of paired samples *t*-tests were conducted to examine the effect of caffeine on perceived confidence at each time point, revealing no significant effects of treatment (Table 1, *p* > 0.16; *g* < 0.42 in each case).

#### 3.2.2. Flanker Task

**Figure 4:**
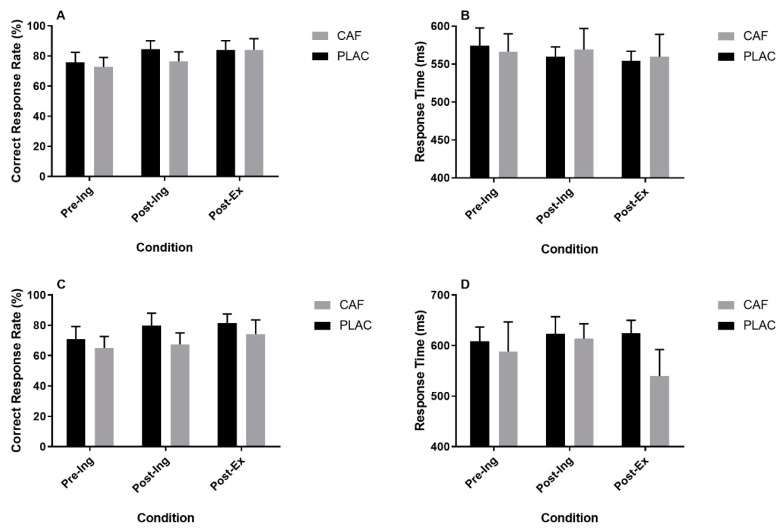
The effect of caffeine consumption on correct response rate (CRR) and response time (RT) for congruent (**A**,**B**) and incongruent (**C**,**D**) stimuli assessed using a Flanker task. Data represented as mean ± S.E.M.; *n* = 12 in each case; Pre-Ing = pre-ingestion; Post-Ing = post-ingestion; Post-Ex = post-exercise; CAF = Caffeine; PLAC = Placebo.

CRR and RT for both congruent and incongruent tasks were not significantly affected by treatment (Figure 4, ANOVA *p* > 0.11; *ηp*^2^ < 0.22 in each case) or time (Figure 4, ANOVA *p* > 0.23; *ηp*^2^ < 0.13 in each case), nor was there a significant treatment × time interaction (Figure 4, ANOVA *p* > 0.19; *ηp*^2^ < 0.14 in each case).

## 4. Discussion

The present study provides the most comprehensive analysis of the effects of acute caffeine consumption on the standing and dynamic balance performance of older adults. Furthermore, the present work sought to examine if the interaction between standing and dynamic balance in combination with a cognitive dual task could be modulated by caffeine consumption. Despite the reported benefit of caffeine on muscular strength [19,28] and cognitive function [9], both important facets of balance performance, data obtained in the present study indicated that caffeine elicited little benefit on measures of static and dynamic balance. In fact, some measures of bipedal standing balance were worsened by caffeine consumption. Despite the introduction of a cognitive dual task influencing both balance and cognitive performance in an assessment-specific manner, this interaction was not modulated by acute caffeine consumption. These findings in part agree with our hypothesis, whereby caffeine reduced balance performance during bipedal standing, but they refute the idea that acute caffeine ingestion can improve dynamic balance and performance during the completion of a cognitive dual task.

### 4.1. The Effect of Caffeine on Static and Dynamic Balance

Data obtained in the present study add weight to the limited quantity of evidence and ambiguous findings of previous work examining the effect of acute caffeine consumption on the physical performance of older adults [19,20,21,22,28]. Similar to work by Jensen, Norager, Fenger-Gron, Weimann, Moller, Madsen and Laurberg [21] and Tallis, Duncan, Wright, Eyre, Bryant, Langdon and James [22] measuring muscular strength, these results indicate no beneficial effects of caffeine on measures of standing and dynamic balance in older adults. Given the limited quantity of published work specifically examining caffeine’s effect on balance, it is difficult to contextualise these findings with respect to previous data. In line with the small body of previous work [27,28], the findings of the current study would appear to support the idea that acute caffeine consumption may be detrimental to standing balance performance in older adults. However, Swift and Tiplady [27] demonstrated that anterior-posterior displacement on the centre of mass was greater following caffeine ingestion and only 3 h post-consumption. Conversely, COP_AP_ was unaffected in the present study whilst other measures of standing balance performance (COP_ML_, COP_Path_, COP_Velocity_) were affected ~45 min post-ingestion. This discrepancy may be a result of differences in caffeine concentration, whereby Swift and Tiplady [27] administered caffeine as an absolute dose, whilst a relative dose was used in the present work. The current data indicate for the first time that caffeine ingestion may increase medial-lateral sway during standing balance task, a metric more important for identifying fall risk in older adults [12]. Furthermore, these data indicate that such detrimental effects of caffeine on standing balance performance occur in conditions of reduced visual and somatosensory input, as indicated by the similar trend in performance during eyes closed and standing on compliant foam conditions. Thus, acute caffeine may elevate fall risk in older adults with visual and somatosensory impairments, where balance is already compromised.

A mechanistic justification as to why caffeine might increase some measures of postural sway may be related to caffeine’s effect as a stimulant. It has been reported that caffeine can cause jitters and irritability [69], which may manifest in ambulation in the body’s centre of mass during quiet standing. Furthermore, there is evidence to suggest that caffeine may have a stimulatory effect on respiration [70], with changes in the pattern of respiration also likely to cause greater ambulation of the body’s centre of mass [71].

The present study uniquely examined the effect of acute caffeine ingestion on measures of dynamic balance in older adults. The data indicate that caffeine failed to elicit any effect on the outcome measures assessed across the various dynamic balance assessments. This finding would appear to contradict previous work by [23] that demonstrated that 6 mg·kg^−1^ caffeine ingestion improved some measures of postural control in young adult males during unipedal dynamic balance assessments of the dominant limb. Beyond differences in population, age and dose of caffeine administered when compared to the present work, Kara, Patlar, Stoffregen and Erkmen [23] measured dynamic balance in a unipedal standing task on a moving platform. This is somewhat different to the range of dynamic balance assessments used in the present study, where the demand on the balance control systems is likely to differ.

Such limited effects of caffeine ingestion on measures of dynamic balance used in the present study may come as a surprise given that it has commonly been cited that the balance performance in older adults is comprised by the age-related reduction in muscular strength [13,14]. Whilst it is anticipated that the demand on muscular strength and power would be much greater in dynamic balance tasks compared to that during bipedal quiet standing, these results imply that the caffeine dose used in the present study was not sufficient in inducing an increase in muscular strength, or that the balance performance of the older adults assessed in the current study was not limited by the contractile performance of skeletal muscle. Whilst it may not be possible to exclude either of these factors, the elevated caffeine-induced increased RFD, demonstrated in the force-time assessment of sit-to-stand performance, may be a proxy for elevated muscular power, though this may not be at the level to improve dynamic balance performance.

Caffeine failed to elicit an effect on TUG performance; this would appear to directly contradict previous work by Duncan, Clarke, Tallis, Guimaraes-Ferreira and Leddington Wright [19], who demonstrated that 3 mg·kg^−1^ caffeine ingestion improved TUG performance in an older adult sample. Given the similarities in population and in methodological approach, the rationale for these differences is unclear. The discrepancy may be related to differences in the physical activity levels of the population, differences in caffeine consumption habits or the multifaceted nature of successful TUG performance.

### 4.2. The Effect of Caffeine on Cognitive Dual Task Processing

Ingestion of 3 mg·kg^−1^ caffeine failed to modulate the interaction between performance on the balance task during the simultaneous completion of a cognitive dual task. This result may be somewhat surprising given the quantity of literature that outlines the positive effects of caffeine on cognitive function (see review [9]). Furthermore, there is published literature denoting the positive effects of caffeine ingestion on cognitive dual task performance [72,73], though evidence specifically in older adults examining the influence of caffeine on simultaneous completion of a motor and cognitive task is lacking.

It is well established that the simultaneous execution of a secondary task places high demands on the information processing system [72], and evidence suggests that cognitive dual task performance is impaired with age [34,35], causing limited mobility and increasing fall risk [36]. When considered holistically, the present results indicate that when both balance and cognitive tasks are completed in synergy, performance is reduced. However, the response was not uniform across the different balance assessments. During the bipedal standing task, the introduction of a cognitive dual task had no effect on balance performance in the EO condition, whilst performance in the EOF condition was improved. However, this came at the expense of performance in the serial 3s counting task. It is interesting to note that performance in the serial 7s task was unaffected, which is surprising given that it was anticipated that this would result in a greater cognitive challenge and thus demand greater information processing capacity. Conversely, both dynamic balance and cognitive function were impaired when the counting tasks were completed simultaneously with the YBT-LQ. With respect to the dual task paradigm, it is generally accepted that the greater the cognitive demand, the larger the impact [74]. As such, the more substantial effect seen during the dynamic balance assessment is unsurprising.

These results are particularly difficult to contextualise with respect to previous work given that there is evidence to indicate that the completion of a secondary task can impair [32,75,76,77] or improve postural control [33,78], with evidence supporting [33,78] and contradictory to the posture first hypothesis (the idea that posture is hierarchically more important than the performance of these secondary tasks) [75]. What is clear from this body of evidence is that the influence of a cognitive dual task varies depending on task complexity, age and balance ability [79]. Despite the complexity of the dual task interaction, the present data imply that the introduction of a secondary task is detrimental to cognitive performance and dynamic balance. Such effects are likely to be detrimental to performance in real-world tasks of daily living where motor performance is coupled with the completion of cognitive tasks.

### 4.3. Broader Applications, Limitations and Future Work

Whilst it is clear from the data obtained in the present study that caffeine may not be an effective nutritional supplement to yield positive changes in balance performance, it is also unlikely that caffeine would increase the risk of falls, despite the increase in medial-lateral sway in the bipedal standing task. Although elevated medial-lateral sway may present a bigger risk factor for falls [12], caffeine ingestion failed to elicit any changes in dynamic balance performance, a modality that carries an elevated fall risk. As such, the lack of consistency in the caffeine response across static and dynamic assessments of balance brings into question the meaningfulness of caffeine-induced changes in bipedal static balance. Although caffeine failed to modulate the interaction between balance performance whilst simultaneously completing a cognitive dual task, this combination of activities may have negative consequences for the completion of activities of daily living.

Although yet to be established in older adults, there is evidence to suggest that the erogenicity of caffeine may be influenced by time of day, with a greater response for measures of physical performance in the morning compared to late afternoon/evening [80,81,82]. Although it was not possible to determine this from the present study, based on this previous evidence, these data likely correspond with the optimal time course of caffeine’s effect, and thus, caffeine-induced responses may be less prevalent later in the day. Evidence exploring the time of day effects of caffeine on facets of cognitive function is sparse, but there is some work to suggest that effects may be more pronounced in the morning [83]. To the author’s knowledge, the effect of caffeine on cognitive dual-tasking has not been examined.

Although this work offers a valuable insight into the effects of acute caffeine ingestion on the balance performance of older adults, the study is not without limitation. Caution should be exercised with respect to the interpretation of these data as these results may only be generalisable to active older adults who demonstrate high balance confidence. Future work should consider examining the effects of caffeine on the balance and functional performance of older, frail participants. Potentially, the outcomes would differ to those of the present study, given that frail older adults are likely to have greater fall risk, which in part is attributed to further compromised muscular function. The current work is also limited in that it only considers the effect of caffeine on balance performance after a period of rest. There is published evidence to suggest that balance performance is compromised following a period of exercise [49], indicating an elevated fall risk following a period of physical activity. Beyond potentially increasing the performance of fatigued muscle, caffeine consumed prior to exercise may reduce the fatigue effects, thus working to reduce the effects of exercise on balance performance.

Despite the sample size being representative [7,22,84,85,86] and, in many cases, greater [41,87,88,89,90,91,92] than that used in previous work examining the ergogenic effects of caffeine, a limitation of the present work, and prevalent across the published literature, is low statistical power. A reliance on estimates of effect size and more traditional null hypothesis testing helps to reduce this concern. Recruiting older adults for such studies is challenging given contraindications to participation that are less prevalent in younger adults. Whilst this work offers an appropriate first stage for scientific scrutiny of the issue of caffeine effects on balance, future work should consider measuring these effects in a larger sample.

Trends in typical daily caffeine consumption have been shown to be consistent between the third and sixth decade before reducing in older age groups [93,94]. As such, given the age of the participants in the present study, it is reasonable to suggest that habituation to caffeine’s effects may have influenced the responses. Despite being a point of contention [95,96], there is evidence in younger adult populations to indicate that continued exposure to caffeine may dampen its performance-enhancing effects [97,98]. Whilst a 3 mg·kg^−1^ dose is important to investigate given that it closely represents the typical caffeine consumption habits of this population and is more likely achievable without the need for anhydrous caffeine, it is proposed that a dose greater than that typically consumed might be an effective strategy to overcome the potential effect of habituation [99]. An elevated dose may therefore evoke more pronounced effects than those seen in the present study, but results from studies using higher caffeine doses may be limited in their generalisability to the older adult population.

There is also evidence to suggest that an individual’s sensitivity to caffeine and speed of metabolism may be influenced by ADORA2A and CYP1A2 single-nucleotide polymorphisms, respectively [100,101,102]. This was not assessed in the present study and, to the authors’ knowledge, has not been investigated in an older adult population. This may present an interesting area of future work given a potential interaction between genetic predisposition and habituation to caffeine’s effects.

## 5. Conclusions

The results of the present study indicate that acute caffeine ingestion negatively affects the bipedal standing balance of older adults but has limited effects on dynamic balance performance and balance performance when completed simultaneously with a cognitive dual task. Given that caffeine failed to elicit a change in dynamic balance, it is unlikely that the caffeine-induced increase in postural sway seen in the bipedal standing balance assessment induces an elevated fall risk. Caffeine ingestion did however evoke an elevated rate of force development when the force-time characteristics of sit-to-stand performance were analysed; this may be indicative of an increase in muscular power. Irrespective of caffeine ingestion, the introduction of a cognitive dual task had negative implications for performance. During the dual task trials, balance was unaffected or improved in the bipedal standing tasks but reduced in the YBT-LQ assessment. In both cases, there was evidence for a reduction in the performance on the cognitive task. In sum, these data indicate that dual task processing may have negative consequences for activities of daily living whereby older adults are expected to perform motor tasks in combination with cognitive tasks. Future work is needed to establish whether caffeine can modulate the effect of exercise on the balance performance of older adults.

## Figures and Tables

**Table 1:** The effect of caffeine on BRUMS subscales.

		Pre-Ingestion	Post-Ingestion	Post-Exercise
Anger	PLAC	0.0 ± 0.0	0.0 ± 0.0	0.3 ± 1.2
	CAF	0.0 ± 0.0	0.0 ± 0.0	0.4 ± 1.2
Confidence	PLAC	0.3 ± 0.8	0.3 ± 0.8	0.0 ± 0.0
	CAF	0.1 ± 0.3	0.1 ± 0.3	0.3 ± 0.8
Depression	PLAC	0.0 ± 0.0	0.0 ± 0.0	0.0 ± 0.0
	CAF	0.0 ± 0.0	0.0 ± 0.0	0.2 ± 0.6
Fatigue	PLAC	0.3 ± 0.7	0.3 ± 0.7	0.1 ± 0.3
	CAF	0.8 ± 1.9	0.3 ± 0.7	0.6 ± 1.2
Tension	PLAC	0.7 ± 1.1	0.4 ± 0.8	0.3 ± 1.2
	CAF	0.8 ± 1.5	0.5 ± 1.2	0.2 ± 0.6
Vigour	PLAC	8.2 ± 3.7	7.7 ± 4.2	7.8 ± 4.2
	CAF	9.0 ± 4.0	8.7 ± 4.5	9.0 ± 5.3

Data represented as mean ± S.E.M.; *n* = 12 in each case; BRUMS = Brunel Mood Scale; CAF = Caffeine; PLAC = Placebo.

## References

[1] Graham T.E., Rush J.W.E., Van Soeren M.H. (1994). Caffeine and Exercise: Metabolism and Performance. Can. J. Appl. Physiol..

[2] Tallis J., Duncan M.J., James R.S. (2015). What can isolated skeletal muscle experiments tell us about the effects of caffeine on exercise performance?. Br. J. Pharmacol..

[3] Davis J.K., Green J.M. (2009). Caffeine and Anaerobic Performance. Sports Med..

[4] Magkos F., Kavouras S.A. (2005). Caffeine Use in Sports, Pharmacokinetics in Man, and Cellular Mechanisms of Action. Crit. Rev. Food Sci. Nutr..

[5] Grgic J., Mikulic P., Schoenfeld B.J., Bishop D.J., Pedisic Z. (2018). The Influence of Caffeine Supplementation on Resistance Exercise: A Review. Sports Med..

[6] Grgic J., Grgic I., Pickering C., Schoenfeld B.J., Bishop D.J., Pedišić Ž. (2020). Wake up and smell the coffee: Caffeine supplementation and exercise performance—An umbrella review of 21 published meta-analyses. Br. J. Sports Med..

[7] Southward K., Rutherfurd-Markwick K.J., Ali A. (2018). The Effect of Acute Caffeine Ingestion on Endurance Performance: A Systematic Review and Meta–Analysis. Sports Med..

[8] Astorino T.A., Roberson D.W. (2010). Efficacy of Acute Caffeine Ingestion for Short-term High-Intensity Exercise Performance: A Systematic Review. J. Strength Cond. Res..

[9] Nehlig A. (2010). Is Caffeine a Cognitive Enhancer?. J. Alzheimer’s Dis..

[10] Johansson J., Nordström A., Gustafson Y., Westling G., Nordström P. (2017). Increased postural sway during quiet stance as a risk factor for prospective falls in community-dwelling elderly individuals. Ageing.

[11] WHO Falls. http://www.who.int/mediacentre/factsheets/fs344/en/.

[12] Melzer I., Benjuya N., Kaplanski J. (2004). Postural stability in the elderly: A comparison between fallers and non-fallers. Age Ageing.

[13] Lee I.-H., Park S.-Y. (2013). Balance Improvement by Strength Training for the Elderly. J. Phys. Ther. Sci..

[14] Benichou O., Lord S.R. (2016). Rationale for Strengthening Muscle to Prevent Falls and Fractures: A Review of the Evidence. Calcif. Tissue Int..

[15] Yang N.-P., Hsu N.-W., Lin C.-H., Chen H.-C., Tsao H.-M., Lo S.-S., Chou P. (2018). Relationship between muscle strength and fall episodes among the elderly: The Yilan study, Taiwan. BMC Geriatr..

[16] Van Ancum J.M., Pijnappels M., Jonkman N.H., Scheerman K., Verlaan S., Meskers C.G.M., Maier A.B. (2018). Muscle mass and muscle strength are associated with pre- and post-hospitalization falls in older male inpatients: A longitudinal cohort study. BMC Geriatr..

[17] Warren G.L., Park N.D., Maresca R.D., McKibans K.I., Millard-Stafford M.L. (2010). Effect of Caffeine Ingestion on Muscular Strength and Endurance. Med. Sci. Sports Exerc..

[18] Grgic J., Pickering C. (2019). The effects of caffeine ingestion on isokinetic muscular strength: A meta-analysis. J. Sci. Med. Sport.

[19] Duncan M.J., Clarke N.D., Tallis J., Guimarães-Ferreira L., Wright S.L. (2014). The effect of caffeine ingestion on functional performance in older adults. J. Nutr. Health Aging.

[20] Momsen A.H., Jensen M.B., Norager C.B., Madsen M.R., Lindholt J.S., Vestersgaard-Andersen T. (2010). Randomized double-blind placebo-controlled crossover study of caffeine in patients with intermittent claudication. BJS.

[21] Jensen M.B., Norager C.B., Fenger-Grøn M., Weimann A., Møller N., Madsen M.R., Laurberg S. (2011). Caffeine Supplementation Had No Effect on Endurance Capacity in Elderly Subjects Who Had Abstained from Caffeine-Containing Nutrition for 8 Hours. J. Caffeine Res..

[22] Tallis J., Duncan M.J., Wright S.L., Eyre E.L.J., Bryant E., Langdon D., James R.S. (2013). Assessment of the ergogenic effect of caffeine supplementation on mood, anticipation timing, and muscular strength in older adults. Physiol. Rep..

[23] Kara M., Patlar S., Stoffregen T.A., Erkmen N. (2018). Effect of caffeine on standing balance during perceptual-cognitive tasks. MoHE.

[24] Franks H.M., Hagedorn H., Hensley V.R., Hensley W.J., Starmer G.A. (1975). The effect of caffeine on human performance, alone and in combination with ethanol. Psychopharmacologia.

[25] Enriquez A., Sklaar J., Viirre E., Chase B. (2009). Effects of caffeine on postural stability. Int. Tinnitus J..

[26] Liguori A., Robinson J.H. (2001). Caffeine antagonism of alcohol-induced driving impairment. Drug Alcohol Depend..

[27] Swift C.G., Tiplady B. (1988). The effects of age on the response to caffeine. Psychopharmacologia.

[28] Norager C.B., Jensen M.B., Madsen M.R., Laurberg S. (2005). Caffeine improves endurance in 75-yr-old citizens: A randomized, double-blind, placebo-controlled, crossover study. J. Appl. Physiol..

[29] Kiss R., Schedler S., Muehlbauer T. (2018). Associations Between Types of Balance Performance in Healthy Individuals Across the Lifespan: A Systematic Review and Meta-Analysis. Front. Physiol..

[30] A Talbot L., Musiol R.J., Witham E.K., Metter E.J. (2005). Falls in young, middle-aged and older community dwelling adults: Perceived cause, environmental factors and injury. BMC Public Health.

[31] Matson T., Schinkel-Ivy A. (2020). How does balance during functional tasks change across older adulthood?. Gait Posture.

[32] Bergamin M., Gobbo S., Ezanotto T., Sieverdes J.C., Alberton C.L., Zaccaria M., Ermolao A. (2014). Influence of age on postural sway during different dual-task conditions. Front. Aging Neurosci..

[33] Resch J.E., May B., Tomporowski P.D., Ferrara M.S. (2011). Balance Performance with a Cognitive Task: A Continuation of the Dual-Task Testing Paradigm. J. Athl. Train..

[34] Al-Yahya E., Dawes H., Smith L., Dennis A., Howells K., Cockburn J. (2011). Cognitive motor interference while walking: A systematic review and meta-analysis. Neurosci. Biobehav. Rev..

[35] Ruffieux J., Keller M., Lauber B., Taube W. (2015). Changes in Standing and Walking Performance Under Dual-Task Conditions Across the Lifespan. Sports Med..

[36] Plummer-D’Amato P., Zukowski L.A., Giuliani C., Hall A.M., Zurakowski D. (2015). Effects of Physical Exercise Interventions on Gait-Related Dual-Task Interference in Older Adults: A Systematic Review and Meta-Analysis. Gerontology.

[37] Maughan R.J. (1999). Nutritional ergogenic aids and exercise performance. Nutr. Res. Rev..

[38] Powell L.E., Myers A.M. (1995). The Activities-specific Balance Confidence (ABC) Scale. J. Gerontol. Ser. A.

[39] Terry P.C., Lane A.M. (2000). Development of normative data for the profile of mood states for use with athletic samples. J. Appl. Sports Psychol..

[40] Duncan M.J., Dobell A.P., Caygill C.L., Eyre E., Tallis J., Dobell A. (2019). The effect of acute caffeine ingestion on upper body anaerobic exercise and cognitive performance. Eur. J. Sport Sci..

[41] Santos R.D.A., Kiss M.A.P.D.M., Silva-Cavalcante M.D., Correia-Oliveira C.R., Bertuzzi R., Bishop D., Lima-Silva A. (2013). Caffeine Alters Anaerobic Distribution and Pacing during a 4000-m Cycling Time Trial. PLoS ONE.

[42] Doherty M., Smith P.M. (2004). Effects of Caffeine Ingestion on Exercise Testing: A Meta-Analysis. Int. J. Sport Nutr. Exerc. Metab..

[43] Bell D.G., McLellan T.M. (2002). Exercise endurance 1, 3, and 6 h after caffeine ingestion in caffeine users and nonusers. J. Appl. Physiol..

[44] Juliano L.M., Griffiths R.R. (2004). A critical review of caffeine withdrawal: Empirical validation of symptoms and signs, incidence, severity, and associated features. Psychopharmacologia.

[45] McLellan T.M., Caldwell J.A., Lieberman H.R. (2016). A review of caffeine’s effects on cognitive, physical and occupational performance. Neurosci. Biobehav. Rev..

[46] Verster J.C., Koenig J. (2018). Caffeine intake and its sources: A review of national representative studies. Crit. Rev. Food Sci. Nutr..

[47] Teo W., Newton M.J., McGuigan M.R. (2011). Circadian Rhythms in Exercise Performance: Implications for Hormonal and Muscular Adaptation. J. Sports Sci. Med..

[48] Donath L., Van Dieën J., Faude O. (2016). Exercise-based fall prevention in the elderly: What about agility?. Sports Med..

[49] Hill M.W., Oxford S.W., Duncan M.J., Price M.J. (2015). The effects of arm crank ergometry, cycle ergometry and treadmill walking on postural sway in healthy older females. Gait Posture.

[50] Pinsault N., Vuillerme N. (2009). Test–retest reliability of centre of foot pressure measures to assess postural control during unperturbed stance. Med. Eng. Phys..

[51] Shaffer S.W., Teyhen D.S., Lorenson C.L., Warren R.L., Koreerat C.M., Straseske C.A., Childs J.D. (2013). Y-Balance Test: A Reliability Study Involving Multiple Raters. Mil. Med..

[52] Tiedemann A., Shimada H., Sherrington C., Murray S., Lord S. (2008). The comparative ability of eight functional mobility tests for predicting falls in community-dwelling older people. Age Ageing.

[53] Rikli R.E., Jones C.J. (2013). Development and Validation of Criterion-Referenced Clinically Relevant Fitness Standards for Maintaining Physical Independence in Later Years. Gerontology.

[54] Beauchet O., Fantino B., Allali G., Muir S.W., Monteroodasso M., Annweiler C. (2011). Timed up and go test and risk of falls in older adults: A systematic review. J. Nutr. Health Aging.

[55] Whitney S.L., Marchetti G.F., Morris L.O., Sparto P.J. (2007). The Reliability and Validity of the Four Square Step Test for People With Balance Deficits Secondary to a Vestibular Disorder. Arch. Phys. Med. Rehabil..

[56] Bristow T., Jih C.-S., Slabich A., Gunn J. (2016). Standardization and adult norms for the sequential subtracting tasks of serial 3’s and 7’s. Appl. Neuropsychol. Adult.

[57] Baetens T., De Kegel A., Palmans T., Oostra K., Vanderstraeten G., Cambier D. (2013). Gait Analysis With Cognitive-Motor Dual Tasks to Distinguish Fallers from Nonfallers Among Rehabilitating Stroke Patients. Arch. Phys. Med. Rehabil..

[58] Wild L.B., De Lima D.B., Balardin J.B., Rizzi L., Giacobbo B.L., Oliveira H.B., Argimon I.I.D.L., Peyré-Tartaruga L.A., Rieder C.R.M., Bromberg E. (2013). Characterization of cognitive and motor performance during dual-tasking in healthy older adults and patients with Parkinson’s disease. J. Neurol..

[59] Eriksen B.A., Eriksen C.W. (1974). Effects of noise letters upon the identification of a target letter in a nonsearch task. Percept. Psychophys..

[60] Davelaar E.J., Stevens J. (2009). Sequential dependencies in the Eriksen flanker task: A direct comparison of two competing accounts. Psychon. Bull. Rev..

[61] Mathôt S., Schreij D., Theeuwes J. (2011). OpenSesame: An open-source, graphical experiment builder for the social sciences. Behav. Res. Methods.

[62] Jaijee S.K., Quinlan M., Tokarczuk P., Clemence M., Howard L.S., Gibbs J.S.R., O’Regan D.P. (2018). Exercise cardiac MRI unmasks right ventricular dysfunction in acute hypoxia and chronic pulmonary arterial hypertension. Am. J. Physiol. Circ. Physiol..

[63] Blanca M.J., Alarcón R., Arnau J., Bono R., Bendayan R. (2017). Non-normal data: Is ANOVA still a valid option?. Psicothema.

[64] Richardson J.T. (2011). Eta squared and partial eta squared as measures of effect size in educational research. Educ. Res. Rev..

[65] Lakens D. (2013). Calculating and reporting effect sizes to facilitate cumulative science: A practical primer for t-tests and ANOVAs. Front. Psychol..

[66] Ivarsson A., Andersen M.B., Johnson U., Lindwall M. (2013). To adjust or not adjust: Nonparametric effect sizes, confidence intervals, and real-world meaning. Psychol. Sport Exerc..

[67] Hopkins W.G., Marshall S.W., Batterham A.M., Hanin J. (2009). Progressive Statistics for Studies in Sports Medicine and Exercise Science. Med. Sci. Sports Exerc..

[68] Cohen J. (2013). Statistical Power Analysis for the Behavioral Sciences.

[69] Burke L., Desbrow B., Spriet L.L. (2013). Caffeine for Sports Performance. Caffeine Sports Perform..

[70] Chapman R.F., Mickleborough T.D. (2009). The Effects of Caffeine on Ventilation and Pulmonary Function During Exercise: An Often-Overlooked Response. Physician Sportsmed..

[71] Hodges P.W., Gurfinkel V.S., Brumagne S., Smith T.C., Cordo P.C. (2002). Coexistence of stability and mobility in postural control: Evidence from postural compensation for respiration. Exp. Brain Res..

[72] Van Duinen H., Lorist M.M., Zijdewind I. (2005). The effect of caffeine on cognitive task performance and motor fatigue. Psychopharmacologia.

[73] Brice C.F., Smith A.P. (2002). Effects of caffeine on mood and performance: A study of realistic consumption. Psychopharmacologia.

[74] Day B.L., Lord S.R. (2018). Balance, Gait, and Falls.

[75] Shumway-Cook A., Woollacott M., Kerns K.A., Baldwin M. (1997). The Effects of Two Types of Cognitive Tasks on Postural Stability in Older Adults With and Without a History of Falls. J. Gerontol. Ser. A Boil. Sci. Med Sci..

[76] Yardley L., Gardner M., Leadbetter A., Lavie N. (1999). Effect of articulatory and mental tasks on postural control. NeuroReport.

[77] Ceyte H., Lion A., Caudron S., Kriem B., Perrin P.P., Gauchard G.C. (2014). Does calculating impair postural stabilization allowed by visual cues?. Exp. Brain Res..

[78] Broglio S.P., Tomporowski P.D., Ferrara M.S. (2005). Balance Performance with a Cognitive Task: A Dual-Task Testing Paradigm. Med. Sci. Sports Exerc..

[79] Brauer S.G., Woollacott M., Shumway-Cook A. (2001). The Interacting Effects of Cognitive Demand and Recovery of Postural Stability in Balance-Impaired Elderly Persons. J. Gerontol. Ser. A Boil. Sci. Med Sci..

[80] Mora-Rodriguez R., Pallarés J.G., López-Gullón J.M., López-Samanes Á., Fernández-Elías V.E., Ortega J.F. (2015). Improvements on neuromuscular performance with caffeine ingestion depend on the time-of-day. J. Sci. Med. Sport.

[81] Boyett J.C., Giersch G.E.W., Womack C.J., Saunders M.J., Hughey C.A., Daley H.M., Luden N.D. (2016). Time of Day and Training Status Both Impact the Efficacy of Caffeine for Short Duration Cycling Performance. Nutrients.

[82] Pataky M.W., Womack C.J., Saunders M.J., Goffe J.L., D’Lugos A.C., Elsohemy A., Luden N.D. (2016). Caffeine and 3-km cycling performance: Effects of mouth rinsing, genotype, and time of day. Scand. J. Med. Sci. Sports.

[83] Sherman S.M., Buckley T.P., Baena E., Ryan L. (2016). Caffeine Enhances Memory Performance in Young Adults during Their Non-optimal Time of Day. Front. Psychol..

[84] Doherty M., Smith P.M. (2005). Effects of caffeine ingestion on rating of perceived exertion during and after exercise: A meta-analysis. Scand. J. Med. Sci. Sports.

[85] Grgic J., Trexler E.T., Lazinica B., Pedišić Ž. (2018). Effects of caffeine intake on muscle strength and power: A systematic review and meta-analysis. J. Int. Soc. Sports Nutr..

[86] Souissi M., Abedelmalek S., Chtourou H., Atheymen R., Hakim A., Sahnoun Z. (2012). Effects of Morning Caffeine’ Ingestion on Mood States, Simple Reaction Time, and Short-Term Maximal Performance on Elite Judoists. Asian J. Sports Med..

[87] A Astorino T., Cottrell T., Lozano A.T., Aburto-Pratt K., Duhon J. (2012). Increases in cycling performance in response to caffeine ingestion are repeatable. Nutr. Res..

[88] Williams A.D., Cribb P.J., Cooke M., Hayes A. (2008). The Effect of Ephedra and Caffeine on Maximal Strength and Power in Resistance-Trained Athletes. J. Strength Cond. Res..

[89] Wallman K.E., Goh J.W., Guelfi K.J. (2010). Effects of Caffeine on Exercise Performance in Sedentary Females. J. Sports Sci. Med..

[90] Andrade-Souza V.A., Bertuzzi R., De Araujo G.G., Bishop D., Lima-Silva A. (2015). Effects of isolated or combined carbohydrate and caffeine supplementation between 2 daily training sessions on soccer performance. Appl. Physiol. Nutr. Metab..

[91] Clarke J.S., Highton J.M., Close G.L., Twist C. (2019). Carbohydrate and Caffeine Improves High-Intensity Running of Elite Rugby League Interchange Players During Simulated Match Play. J. Strength Cond. Res..

[92] Hurley C.F., Hatfield D.L., Riebe D. (2013). The Effect of Caffeine Ingestion on Delayed Onset Muscle Soreness. J. Strength Cond. Res..

[93] Fulgoni V.L., Keast D.R., Lieberman H.R. (2015). Trends in intake and sources of caffeine in the diets of US adults: 2001–2010. Am. J. Clin. Nutr..

[94] Drewnowski A., Rehm C.D. (2016). Sources of Caffeine in Diets of US Children and Adults: Trends by Beverage Type and Purchase Location. Nutrients.

[95] Gonçalves L.D.S., Painelli V.D.S., Yamaguchi G., De Oliveira L.F., Saunders B., Da Silva R.P., Maciel E., Artioli G.G., Roschel H., Gualano B. (2017). Dispelling the myth that habitual caffeine consumption influences the performance response to acute caffeine supplementation. J. Appl. Physiol..

[96] Grgic J., Mikulic P. (2020). Acute effects of caffeine supplementation on resistance exercise, jumping, and Wingate performance: No influence of habitual caffeine intake. Eur. J. Sport Sci..

[97] Beaumont R., Cordery P., Funnell M.P., Mears S.A., James L.J., Watson P. (2017). Chronic ingestion of a low dose of caffeine induces tolerance to the performance benefits of caffeine. J. Sports Sci..

[98] Lara B., Ruiz-Moreno C., Salinero J.J., Del Coso J. (2019). Time course of tolerance to the performance benefits of caffeine. PLoS ONE.

[99] Pickering C., Kiely J. (2019). What Should We Do About Habitual Caffeine Use in Athletes?. Sports Med..

[100] Fulton J.L., Dinas P.C., Carrillo A.E., Edsall J.R., Ryan E.J., Ryan E.J. (2018). Impact of Genetic Variability on Physiological Responses to Caffeine in Humans: A Systematic Review. Nutrients.

[101] Grgic J., Pickering C., Del Coso J., Schoenfeld B.J., Mikulic P. (2020). CYP1A2 genotype and acute ergogenic effects of caffeine intake on exercise performance: A systematic review. Eur. J. Nutr..

[102] Southward K., Rutherfurd-Markwick K., Badenhorst C.E., Ali A. (2018). The Role of Genetics in Moderating the Inter-Individual Differences in the Ergogenicity of Caffeine. Nutrients.

